# White matter abnormalities in misophonia

**DOI:** 10.1016/j.nicl.2021.102787

**Published:** 2021-08-21

**Authors:** Nadine Eijsker, Arjan Schröder, Luka C. Liebrand, Dirk J.A. Smit, Guido van Wingen, Damiaan Denys

**Affiliations:** a Amsterdam University Medical Centers, University of Amsterdam, Department of Psychiatry, Amsterdam Neuroscience, Meibergdreef 9, Amsterdam 1105 AZ, the Netherlands; b Amsterdam Brain and Cognition, University of Amsterdam, Nieuwe Achtergracht 129, Amsterdam 1001 NK, the Netherlands; c Amsterdam University Medical Centers, University of Amsterdam, Department of Biomedical Engineering and Physics, Meibergdreef 9, Amsterdam 1105 AZ, the Netherlands

**Keywords:** Misophonia, White matter volume, Diffusion tensor imaging, Probabilistic tractography, Attention

## Abstract

Misophonia is a condition in which specific ordinary sounds provoke disproportionately strong negative affect and physiological arousal. Evidence for neurobiological abnormalities underlying misophonia is scarce. Since many psychiatric disorders show white matter (WM) abnormalities, we tested for both macro and micro-structural WM differences between misophonia patients and healthy controls.

We collected T1-weighted and diffusion-weighted magnetic resonance images from 24 patients and 25 matched controls. We tested for group differences in WM volume using whole-brain voxel-based morphometry and used the significant voxels from this analysis as seeds for probabilistic tractography. After calculation of diffusion tensors, we compared group means for fractional anisotropy, mean diffusivity, and directional diffusivities, and applied tract-based spatial statistics for voxel-wise comparison.

Compared to controls, patients had greater left-hemispheric WM volumes in the inferior fronto-occipital fasciculus, anterior thalamic radiation, and body of the corpus callosum connecting bilateral superior frontal gyri. Patients also had lower averaged radial and mean diffusivities and voxel-wise comparison indicated large and widespread clusters of lower mean diffusivity.

We found both macro and microstructural WM abnormalities in our misophonia sample, suggesting misophonia symptomatology is associated with WM alterations. These biological alterations may be related to differences in social-emotional processing, particularly recognition of facial affect, and to attention for affective information.

## Highlights

• We found micro and macro-structural white matter alterations in misophonia.
• Patients had greater white matter volumes in left frontal cortex.
• Patients had lower averaged radial and mean diffusivities.
• Voxel-wise comparison indicated widespread clusters of lower mean diffusivity.

## 1 Introduction

Misophonia is a newly described condition in which specific ordinary sounds, such as breathing or lip-smacking, provoke disproportionately strong involuntary feelings of anger, anxiety, and/or disgust, with accompanying physiological arousal, such as sweating and increased heart rate, and skeletal muscle responses (see Edelstein et al., 2013, Jager et al., 2020, Jastreboff and Jastreboff, 2014, Dozier et al., 2017 for review; Dozier and Morrison, 2017, Potgieter et al., 2019, Rouw and Erfanian, 2018, Schröder et al., 2013, Schröder et al., 2019). It has been proposed that low-intensity stimuli become conditioned triggers over time, resulting in experience and context-dependent misophonic reactions (Schröder et al., 2013, Jastreboff and Jastreboff, 2014, Dozier, 2015). Prevalence rate estimates vary between 3.2% (Jastreboff and Jastreboff 2014) and 6% (Zhou et al., 2017). Population studies with undergraduate students indicate nearly 20–50% of students reported some misophonia-like sound sensitivity (Naylor et al., 2021, Wu et al., 2014), yet such studies are often subject to response bias (Groves et al., 2004). In only 6% this was associated with clinical impairment (Zhou et al., 2017) and when setting the threshold for clinical significance based on symptom severity generally reported by help-seeking misophonia samples (Jager et al., 2020, Schröder et al., 2019), approximately 12% of undergraduates would report clinically significant symptoms (Naylor et al., 2021). Misophonia symptoms are strongly associated with impairments in work/study, social, and family life (Wu et al., 2014, Zhou et al., 2017). The consistent and distinct pattern of symptoms suggests it might be a discrete mental disorder (Jager et al., 2020, Potgieter et al., 2019, Schröder et al., 2013, Taylor, 2017), yet neurobiological evidence remains scarce.

Neuroimaging studies have used audiovisual and auditory stimuli to provoke symptoms and implicated areas involved in salience attribution and emotional processing, including the anterior insula, the cingulate cortex, ventromedial prefrontal and orbitofrontal cortices, amygdala, and hippocampus (Cerliani and Rouw, 2020, Kumar et al., 2017, Schröder et al., 2019). Also areas responsible for sensory processing and behavioral responses have been implicated (Cerliani and Rouw, 2020). Furthermore, evidence points to a symptom provocation-related worsening of cognitive control on the Stroop task (Daniels et al., 2020), generally abnormal alerting attention (Frank et al., 2020), and a response bias – yet intact response inhibition – on the visual stop signal task (Eijsker et al., 2019). We previously found higher resting-state functional connectivity between the amygdala and cerebellum, as well as within the visual cortex as part of the ventral attention network (Eijsker et al., 2021). This evidence points to patients additionally exhibiting attentional and behavioral abnormalities outside of symptom-provoking contexts. Sensory function as early as pre-attentive auditory processing seems affected, as suggested by aberrant event-related electrophysiological potentials in response to (non-trigger) oddball sounds (Schröder et al., 2014).

Only two studies have considered structural abnormalities in misophonia. In patients compared to controls, we previously found greater gray matter (GM) volume in the right amygdala (Eijsker et al., 2021), whereas Kumar et al. (2017) found greater myelination of ventromedial prefrontal GM. White matter (WM) integrity is associated with global cognition and a range of executive and motor functions (Peters et al., 2012, Vernooij et al., 2009). Therefore, it is not surprising that many psychiatric disorders show WM abnormalities. Functional aberrations like those found in misophonia have been linked to WM structure abnormalities in other disorders. For instance, emotional disorders often show reduced WM volumes, on the macrostructural scale, and integrity, on a microstructural scale (Daniels et al., 2013, Jenkins et al., 2016). Additionally, the poor attentional control found in adolescents with attentional, affective, and behavioral disorders has been linked to decreased WM integrity in WM tracts that differed from those showing such a link in healthy participants (Shafer et al., 2020). Moreover, decreased WM integrity has been linked to abnormal auditory and multisensory processing and inattention in children with autism spectrum disorder and/or sensory processing disorders (Chang et al., 2014, Owen et al., 2013). Therefore, we tested whether misophonia patients show differences in WM structure from healthy subjects.

To test for both macro and micro-structural WM differences between patients and controls, we used T1-weighted magnetic resonance imaging (MRI) and diffusion tensor imaging (DTI), respectively. Volumetric WM changes can be assessed by applying voxel-based morphometry (VBM) to T1-weighted data, which is sensitive to local differences independent of large-scale brain volume (Ashburner and Friston 2001), whereas DTI detects microstructural WM differences, including proxies for integrity (see Assaf and Pasternak 2008 for review). We also tested whether WM structure correlated differently with age between the groups. Since reports on WM structure in misophonia are close-to-none and there is considerable heterogeneity between more well-researched psychopathologies, such as trauma-related, autism-spectrum, and obsessive-compulsive disorders (Daniels et al., 2013, DeRamus and Kana, 2015, van den Heuvel et al., 2009), we opted for an exploratory whole-brain voxel-wise approach.

## 2 Materials & methods

### 2.1 Participants

Twenty-four misophonia patients were recruited from the Amsterdam University Medical Centers (Amsterdam UMC, location Academic Medical Center) outpatient clinic. Patients underwent a standard psychiatric interview in which they were diagnosed on the basis of the criteria postulated by Schröder et al. (2013) by three AMC psychiatrists experienced in diagnosing misophonia. The interview additionally assessed general medical and psychiatric histories. Twenty-five controls, matched on age, sex, and education level, were recruited via advertisements at the Amsterdam UMC and University of Amsterdam.

Potential participants, females and males aged between 18 and 65 years, were assessed with a telephone interview by a psychiatrist (A.S.) not involved in the initial misophonia diagnosis, in which they were asked about possible (additional) misophonia symptoms, psychiatric diagnoses, current and previous health issues, medication use, alcohol or substance use, and handedness. We screened for personality disorders using the Structured Clinical Interview for DSM-IV axis II Disorders (SCID-II; First et al., 1997). Exclusion criteria for all participants included presence of major depression, anxiety disorder, bipolar disorder, psychotic disorder, autism spectrum disorder, substance related disorder, hearing loss, epilepsy, structural central nervous system disorder, stroke within the last year, and MRI contraindications. No hearing tests were applied, because previous extensive testing, including pure tone, speech audiometry, and loudness discomfort levels, of misophonia patients prior to receiving treatment, did not result in any notable hearing problems. An inclusion criterion only for patients was that they experienced anger – a subset also experienced disgust – in reaction to eating sounds and at least three of the following sounds: heavy breathing/sniffling, keyboard typing, chewing, and slurping. One patient reported symptoms with only two out of four sounds, but was still included because of severe symptoms. Patients additionally were required to not have had treatment for their misophonia. Another exception was made for one patient who had received unsuccessful treatment. Two patients had comorbid attention deficit (hyperactivity) disorder, of whom one used methylphenidate (30 mg daily), and another had a borderline personality disorder.

The study was approved by the Medical Ethics Committee of the Amsterdam UMC. All participants were informed about the nature of the experimental procedures and subsequently provided written informed consent prior to inclusion in the study. Current data were obtained in a larger study in which we also collected functional MRI during symptom-provocation (Schröder et al., 2019), performance of a stop signal task (Eijsker et al., 2019) and rest (Eijsker et al., 2021), with a total scanning time of approximately 43 min.

### 2.2 Demographic and clinical characteristics

Using SPSS software version 25 (IBM), we tested for group differences in sex, age, and education level to ensure demographically similar groups. We also tested for group differences in score on the Symptom Checklist (SCL-90; Derogatis et al., 1973), Hamilton Anxiety and Depression Rating Scales (HAM-A; HAM-D; Hamilton, 1959, Hamilton, 1960), and Buss-Perry Aggression Questionnaire subscales and total score (BPAQ; Buss and Perry 1992) to assess differences in clinical characteristics. We used the Amsterdam Misophonia Scale (A-MISO-S; Schröder et al., 2013), which is an adaptation of the Yale-Brown Obsessive-Compulsive Scale (Goodman et al., 1989), to assess misophonia symptom severity. Factor analysis of A-MISO-S data has indicated it to be a unidimensional tool with good internal consistency (Naylor et al., 2021), yet further validation is needed. We compared groups using Chi-squared tests for categorical variables and Welch’s *t*-tests for continuous variables, respectively. Effect sizes for these tests are expressed in Phi coefficient and Cohen’s *d*, respectively. We corrected for testing 8 clinical scores using the Tukey-Ciminera-Heyse (TCH) method with the modification suggested by Sankoh et al. (1997), which takes the covariance of the tested measures into account and is suited when the scores are highly correlated (r >0.5).

### 2.3 MRI data acquisition

Images were acquired on a Philips Ingenia 3.0 T MRI system (Philips Medical Systems, Best, the Netherlands) with a 32-channel head coil. To minimize movement artifacts, participants’ heads were fixed using foam padding and participants were asked to lay as still as possible. We acquired whole-brain anatomical T1-weighted images (3D MP-RAGE, SENSE factor = 2.5, voxel size = 1 mm^3^, TR/TE = 7000/3.2 ms, matrix = 256×256, field of view = 256×240 mm, 180 transverse slices, scanning time 4 min and 14 s, automatic T1 stabilization) and diffusion-weighted images (2D spin-echo using a single shell without multiband acceleration and cardiac gating, SENSE factor = 2, slice thickness = 2 mm, TR/TE = 8015/92 ms, matrix = 112×112, field of view = 224 mm, 60 transverse slices, whole brain coverage) with diffusion gradients applied along 64 (n = 46, scanning time 9 min and 31 s) or 48 (n = 3; 1 control and 2 patients, scanning time 6 min and 59 s) directions (b = 1000 s/mm^2^), and 4 (n = 36) or 1 (n = 13; 7 controls and 6 patients) reference b = 0 image(s). No reverse-phase encoded scans were acquired. All DICOM files were converted to NifTI format using *dcm2niix* (https://github.com/rordenlab/dcm2niix). See Supplementary Table 1 for additional acquisition information.

#### 2.3.1 VBM analysis

T1-weighted images were preprocessed and analyzed in Statistical Parametric Mapping (SPM) version 12 (Wellcome Trust Centre for Neuroimaging, 2014), implemented in Matlab version R2016a (The MathWorks, Inc.). The T1-weighted images were visually inspected and their origin was manually set to the anterior commissure to ensure correct segmentation. Then, they were segmented into GM, WM, and corticospinal fluid (CSF) using rigid-body alignment and SPM’s tissue priors, saving both native and Dartel imported images. Hereafter, WM templates were created using Dartel (Ashburner, 2007, Ashburner and Friston, 2009), which iteratively aligns the data to generate increasingly crisp templates to which the native space images were subsequently spatially normalized. This resulted in WM maps in Montreal Neurological Institute (MNI) space with voxels of 1.5 mm^3^, which were then smoothed with an 8 mm full width at half maximum (FWHM) Gaussian kernel and Jacobian scaled. Total intracranial volume was calculated by summation of GM, WM, and CSF volumes outputted by segmentation.

We added the groups to a general linear model, also adding age, sex, and total intracranial volume as covariates not-of-interest. All voxels with intensities below 20% of the mean voxel intensity were excluded from analysis (absolute threshold masking at 0.2). We separately tested whether the groups had a different relationship of age with a WM volume group × age interaction, independent of overall group effects, and with age demeaned within groups. No global normalization or overall grand mean scaling were applied. Statistical tests were family-wise error (FWE) rate corrected for multiple comparisons at the peak level (*p* < 0.05, two-tailed).

#### 2.3.2 DTI analysis

Diffusion-weighted images were visually inspected and *eddy qc* was used for quality control (Bastiani et al., 2019). No movements exceeded 3 mm of translation or 3 degrees of rotation and the groups did not differ in average (*t*(47) = 0.1, *p* = .93) or maximum (*t*(47) = 0.3, *p* = .77) movement. Using MRtrix 3.0 functions, we denoised the data (*dwidenoise*; Veraart et al., 2016), corrected for Gibbs ringing artefacts (*mrdegibbs*; Kellner et al., 2016), extracted references images (*dwiextract*) and, if multiple were available, averaged them. Then, using functions from the FDT toolbox of FMRIB Software Library version 6.0 (FSL; http://www.fmrib.ox.ac.uk/fsl; Jenkinson et al., 2012), we created a binary brain mask by applying a 10% threshold (*bet*) to the reference images, which we then used to correct for eddy currents and subject motion (*eddy*; Andersson and Sotiropoulos 2016). Weighted least squares diffusion tensor models were subsequently fit at each voxel (*dtifit*), resulting in maps for each subject for diffusivity along the main axis – axial diffusivity (AD), the combined perpendicular axes – radial diffusivity (RD), mean diffusivity (MD), and fractional anisotropy (FA), which describes how strongly oriented diffusivity is. Using the Tract-based Spatial Statistics (TBSS; Smith et al., 2006) toolbox, these maps were aligned to the 1 mm^3^ FMRIB58_FA target provided by FSL, which is in MNI standard space. This was done by the nonlinear registration tool FNIRT (Andersson et al., 2007), which uses a b-spline representation of the registration warp field (Rueckert et al., 1999). A threshold of 0.2 was applied to the average FA map to obtain a binary FA skeleton for use in group comparison. Lastly, subjects’ aligned FA, MD, RD, and AD images were projected onto this skeleton.

First, we used Welch’s *t*-tests to assess whether the means of these DTI measures differed between the groups and we calculated Cohen’s *d*. We corrected for testing 4 DTI measures using the modified TCH method described above. Then, we applied voxel-wise statistical analysis to the FA, MD, RD, and AD maps using permutation testing (FSL’s *PALM*; 10.000 permutations) with sex and age as covariates and Threshold-Free Cluster Enhancement (TFCE), resulting in FWE-corrected *p*-values. Using FSL’s *cluster*, we extracted cluster and peak information from the t-map masked to show only significant voxels. Affected tracts were identified using the JHU White-Matter Tractography Atlas and JHU ICBM-DTI-81 White-Matter Labels (Mori et al., 2005, Wakana et al., 2007, Hua et al., 2008). For visualization of the results, we fattened the results to increase visibility using *tbss_fill*. We also tested whether the groups had a different relationship of age with any DTI measure (group × age interaction, independent of overall group effects, and with age demeaned within groups) again using permutation testing with TFCE. Scans of 3 participants, 1 control and 2 patients, were acquired with diffusion gradients in 48 instead of 64 directions, so we also tested whether excluded these participants affected the results.

**Fig. 1:**
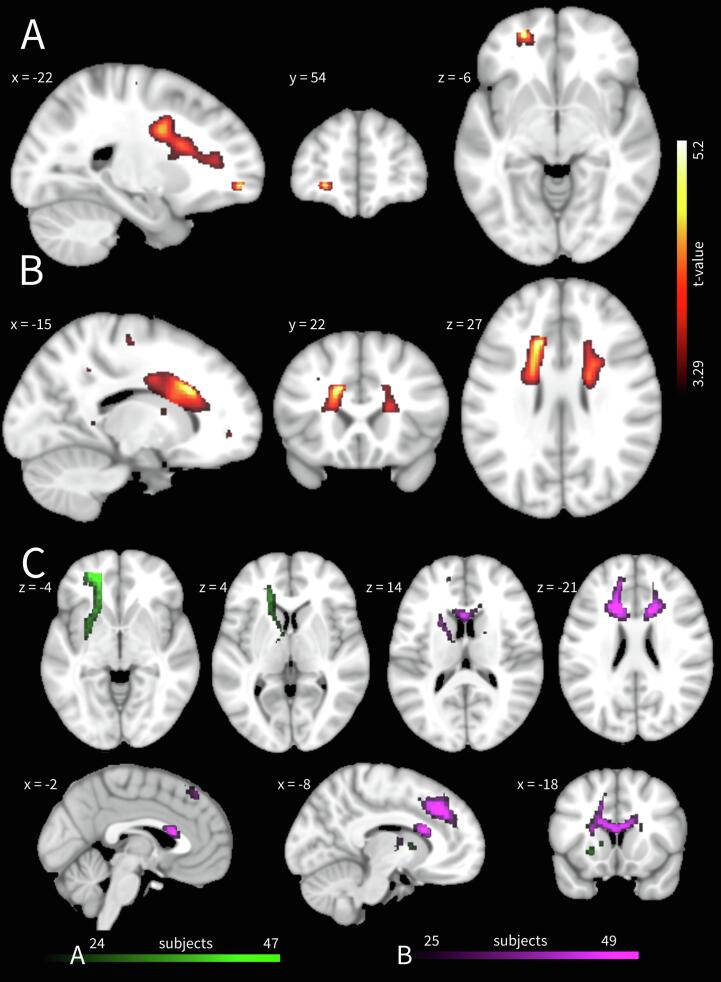
**Patients had greater white matter volumes than controls** at (A) the left inferior fronto-occipital fasciculus/anterior thalamic radiation and (B) the left body of the corpus callosum/anterior thalamic radiation. Voxels with *p*-uncorrected < 0.001 are displayed according to neurological convention. (C) Population map of probabilistic tractography seeded from the white matter volume difference peak voxels depicted in subplot A (green) and B (violet). Only voxels are shown that contained at least 20 (out of 5000) streamlines and that were shared by at least 50% of subjects. (For interpretation of the references to colour in this figure legend, the reader is referred to the web version of this article.)

Lastly, we performed probabilistic tractography using the FDT toolbox (*BEDPOSTX; PROBTRACKX*), which uses Bayesian techniques and Markov Chain Monte Carlo sampling to estimate the most probable location of a pathway (Behrens et al., 2007). Due to a lack of evidence on misophonia WM structure to inform tractography, we did fiber tracking using the peak voxels of the VBM results as seeds. We did not perform tracking on the TBSS results, because these were considerably profuse and diffuse. After transforming the peak voxel MNI coordinates to subject space using SPM’s *coregister* with nearest neighbor interpolation, 5000 streamlines were initiated per seed using a step length of 0.5 mm and a curvature threshold of 0.2. We thresholded these maps to only contain voxels with at least 20 (out of 5000) streamlines and binarized them before transforming them back to MNI space using trilinear interpolation. We inventoried the resulting tracts by identification using the JHU WM Tractography and JHU ICBM-DTI-81 WM labels atlases. For visualization, we then summed and thresholded these maps to only show tracts shared by at least 50% of subjects. For two subjects, tractography using the seed depicted in Fig. 1A did not generate any plausible tracts.

#### 2.3.3 Correlational analysis

To see whether structural abnormalities had a linear relationship with clinical characteristics in patients, we used SPSS to compute Pearson correlations, for patients only, between WM volume at the peak coordinates (based on *p*-value) and mean MD within the FA skeleton and scores on the A-MISOS-S, HAM-A, and anger subscale of the BPAQ. We corrected for testing 3 clinical measures using the Dubey/Armitage-Parmar (D/AP) method with the modification suggested by Sankoh et al. (1997), which is suited when the scores have low correlations (r < 0.5). Lastly, we computed partial correlations to see whether correcting for age and sex substantially influenced the correlations.

## 3 Results

### 3.1 Demographic and clinical characteristics

Table 1 shows group characteristics. The groups did not significantly differ in age, proportion of females/males, or education level. In patients, symptoms emerged on average around the age of twelve and the average symptom severity (A-MISO-S) scored as 14.9 out of a maximum of 40. Patients scored significantly higher than controls on general psychopathy (SCL-90; Derogatis et al., 1973), anxiety (HAM-A), depression (HAM-D), anger (BPAQ total score and subscale), and hate (BPAQ subscale).

**Table 1:** Demographic and clinical characteristics.

	Controls (N = 25)		Patients (N = 24)		Statistical analysis		
					Test statistic	*p*-value	Effect size
Sex (female; N, %)	19 (76%)		18 (75%)		*χ^2^* = 0.007	0.94	0.012
Age (years; mean, SD, range)	33.3 (9.8) 22–56		32.7 (9.5) 18–47		*t*(46.99) *= -*0.22	0.83	−0.062
Educational level (median, range)†	6 (2–7)		6 (2–7)		*χ^2^* = 0.72	0.87	0.122
Age of onset (years; mean, SD)			11.7 (3.3)				

Clinical measures‡	*Mean*	*SD*	*Mean*	*SD*			
A-MISO-S			14.9	2.7			
SCL-90	103.2	13.8	151.05	45.9	*t*(25.96) = 4.83	< 0.001¶	1.425
HAM-A	2.4	3.6	12.9	8.7	*t*(30.78) = 5.51	< 0.001¶	1.589
HAM-D	1.6	2.4	9.5	5.9	*t*(30.48) = 6.09	< 0.001¶	1.768

BPAQ							
Physical Aggression	16.3	3.3	19.5	5.8	*t*(34.25) *=* 2.34	0.026	0.682
Verbal Aggression	11.8	2.9	12.0	2.9	*t*(45.62) *=* 0.14	0.89	0.069
Anger	13.2	3.8	20.4	5.7	*t*(37.72) = 5.08	< 0.001¶	1.492
Hate	14.2	4.0	20.3	8.0	*t*(31.87) = 3.29	0.002¶	0.971
Total score	55.8	9.4	72.1	18.5	*t*(32.05) *=* 3.81	0.001¶	1.118

N = number; SD = standard deviation; A-MISO-S = Amsterdam Misophonia Scale; SCL-90 = Symptom Checklist; HAM-A = Hamilton Anxiety Rating Scale; HAM-D = Hamilton Depression Rating Scale; BPAQ = Bush Perry Aggression Questionnaire.

† Educational level was categorized using the 2011 ISCED system (UNESCO Institute for Statistics, 2012), ranging from 0 (no finished education) to 8 (doctorate obtained).

‡ Missing data: A-MISO-S: 1 patient, SCL90: 1 patient, 2 controls; HAM-A/HAM-D: 2 controls, BPAQ: 1 patient.

¶ Significant after TCH correction with Sankoh et al. (1997) modification (*p* < .009) for testing 8 clinical measures. Effect sizes are expressed in Cohen’s *d* for Welch’s *t*-tests and in Phi coefficient for the Chi-square tests.

### 3.2 White matter volume and probabilistic tractography

Patients had greater WM volumes in frontal white matter (Fig. 1A and B) and we used the two significant peak voxels as seeds for probabilistic tractography. Tractography from the most anterior peak voxel (*t*(44) = 5.17, *p*-FWE = 0.044, MNI [–22, 54, −6]) showed streamlines along the left inferior fronto-occipital fasciculus (IFOF) and anterior thalamic radiation (ATR), whereas tractography from the peak voxel in the body of the corpus callosum (BCC; *t*(44) = 5.12, *p*-FWE = 0.050, MNI [-15, 22, 27]) showed streamlines that extended bilaterally towards the superior frontal gyri (Fig. 1C). Additionally, this seed also showed streamlines along the ATR, yet more along the dorsolateral aspect of the ATR, whereas streamlines from the anterior peak voxel ran more along the ventromedial aspect. We found no group × age interactions for WM volume. See Supplementary Table 2 for an overview of non-significant VBM results.

### 3.3 Diffusivity measures

**Fig. 2:**
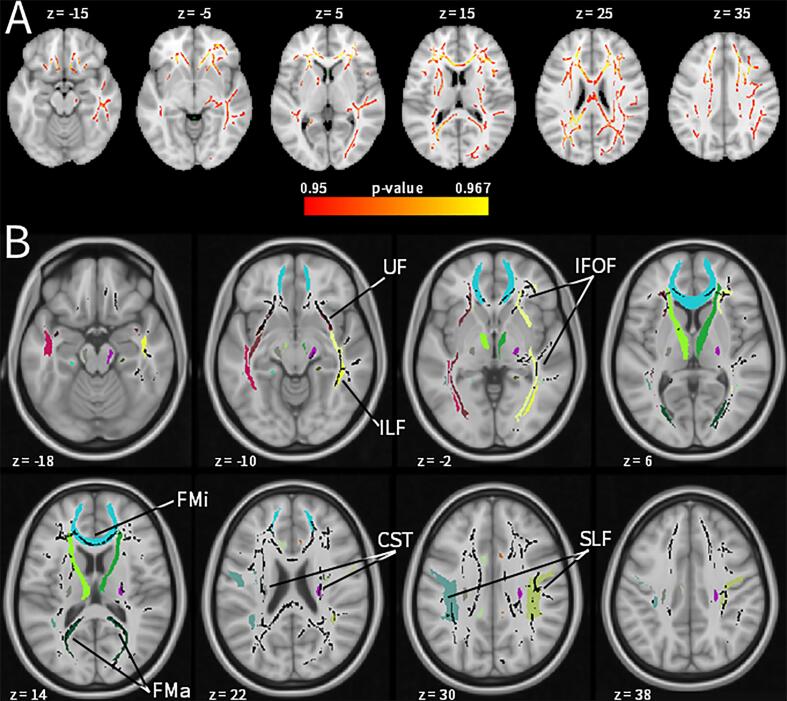
**Patients had widespread lower mean diffusivity than controls.** (A) Voxels showing significantly lower mean diffusivity in patients (red; *p*-FWE < 0.05). (B) Significant voxels (black) displayed on the JHU White-Matter Tractography Atlas. Maps are shown according to neurological convention. ATR = Anterior Thalamic Radiation; ILF = Inferior Longitudinal Fasciculus; UF = Uncinate Fasciculus; IFOF = Inferior Fronto-Occipital Fasciculus; FMi = Forceps Minor; FMa = Forceps Major; CST = Cortico-Spinal Tract; SLF = Superior Longitudinal Fasciculus. (For interpretation of the references to colour in this figure legend, the reader is referred to the web version of this article.)

*T*-testing of FA, MD, RD, and AD averages indicated lower mean MD and RD within the FA skeleton of patients compared to controls, with Cohen’s *d* values around 0.8 (Table 2). Voxel-wise analysis indicated bilateral and widespread lower MD in patients compared to controls, covering the forceps minor/genu of corpus callosum, superior and inferior longitudinal fasciculus, and the inferior fronto-occipital fasciculus (Table 3; Fig. 2). We found no group × age interactions for diffusivity measures. Excluding the 3 scans acquired with different parameters did not render any significant findings non-significant.

**Table 2:** Comparison of DTI measure means between patients and controls.

DTI measure	Controls		Patients		Statistical analysis		
	*Mean*	*SD*	*Mean*	*SD*	Test statistic	*p*-value	Cohen’s *d* [95% CI]
Fractional anisotropy	0.452	0.019	0.462	0.015	*t*(45.03) = -2.09	0.042	0.57 [-0.005 1.137]
Mean diffusivity (*10^−3^)	0.765	0.019	0.750	0.018	*t*(46.98) = 2.80	0.008¶	0.80 [0.216 1.380]
Axial diffusivity (*10^−3^)	1.179	0.023	1.167	0.018	*t*(45.19) = 1.96	0.057	0.56 [-0.015 1.127]
Radial diffusivity (*10^−3^)	0.558	0.022	0.541	0.020	*t*(46.77) = 2.70	0.010¶	0.77 [0.188 1.349]

¶ Significant after TCH correction with Sankoh et al. (1997) modification (*p* < .018) for testing 4 DTI measures.

**Table 3:** Lower voxel-wise mean diffusivity in patients compared to controls.

	Cluster	Peak voxel			
	number of voxels	*p*-value	MNI coordinates		
			x	y	z
R FMi/IFOF/UF/ATR	14,526	0.033	15	28	17
R ILF/IFOF/SLF	6540	0.043	53	−28	−12
L ILF/IFOF/SLF	214	0.050	−40	−36	0
R precuneus white matter	69	0.050	9	−57	23

FMi = Forceps Minor; IFOF = Inferior Fronto-Occipital Fasciculus; UF = Uncinate Fasciculus; ATR = Anterior Thalamic Radiation; ILF = Inferior Longitudinal Fasciculus; SLF = Superior Longitudinal Fasciculus.

### 3.4 Correlational analysis

Neither WM volume at peak voxels (IFOF/ATR nor BCC/ATR) nor peak voxel MD had a linear relationship with the A-MISOS-S, HAM-A, or the anger subscale of the BPAQ (Table 4). Correction for age and sex did not substantially changes these results (Supplementary Table 3).

**Table 4:** Correlations between clinical characteristics and white matter alterations in patients.

Measure	A-MISO-S^a^		HAM-A^b^		Anger (BPAQ)^a^	
	Correlation coefficient	*p*-value	Correlation coefficient	*p*-value	Correlation coefficient	*p*-value
WM IFOF/ATR	0.06	0.780	0.03	0.905	−0.14	0.527
WM BCC/ATR	−0.19	0.377	0.03	0.895	−0.20	0.353
Mean Diffusivity	0.19	0.396	−0.01	0.604	0.07	0.763

No correlations reached the significance threshold (*p* < .018) provided by D/AP correction with Sankoh et al. (1997) modification for testing 3 clinical measures.

A-MISO-S = Amsterdam Misophonia Scale; HAM-A = Hamilton Anxiety Rating Scale; BPAQ = Bush Perry Aggression Questionnaire; WM = White Matter; IFOF = Inferior Fronto-Occipital Fasciculus; ATR = Anterior Thalamic Radiation; BCC = Body of Corpus Callosum.

a N = 23.

b N = 24.

## 4 Discussion

Compared to controls, patients with misophonia had greater white matter (WM) volumes at two locations in the left frontal cortex. Probabilistic tractography using these locations as seeds implicated the inferior fronto-occipital fasciculus (IFOF), anterior thalamic radiation (ATR), and the body of the corpus callosum (BCC). Patients also had lower averaged radial and mean diffusivities (RD and MD, respectively) within the fractional anisotropy (FA) skeleton and voxel-wise comparison indicated large and widespread clusters of lower MD in patients compared to controls.

Macrostructural WM differences were found in tracts connecting fronto-polar and basal orbitofrontal cortex (OFC) to occipital and superior parietal cortex (IFOF; Sarubbo et al., 2013, Wu et al., 2016) and the medial portion of the superior frontal gyri bilaterally (BCC). Both seeds also generated streamlines along the ATR, which connects the OFC and dorsolateral prefrontal cortex (PFC) with the dorsomedial thalamus and anterior thalamic nuclei (ATN; Coenen et al., 2012). On the microstructural level, patients had lower average MD and RD, which scale negatively with myelination and axonal density and positively with presence of other cells, such as astrocytes, and axonal spacing and extracellular volume, respectively (Gulani et al., 2001, Mottershead et al., 2003, Schwartz et al., 2004, Schmierer et al., 2008, Heckel et al., 2015). Logically, an increase in axonal density is accompanied by increased myelin volume and decreased extracellular space/axonal spacing. Since RD does not to correlate with myelin sheath thickness (Schwartz et al., 2004), these results likely reflect high myelin volume as a result of high axonal density in patients (Gulani et al., 2001, Schmierer et al., 2008). The findings also point to high WM integrity, because MD and RD scale negatively with FA and (sensory) nerve conduction velocity, respectively (Gulani et al., 2001, Heckel et al., 2015). We found a medium to large effect size (Cohen’s *d* = 0.6), though statistically insignificant effect, for higher mean FA in patients. FA depends on the ratio of the directional diffusivities, increasing when AD increases and/or RD decreases. Both directional diffusivities were lower rather than higher in patients, explaining why the group difference in MD, and not FA, reached statistical significance. Voxel-wise analysis indicated lower MD for patients compared to controls in various WM tracts, including bilateral inferior fronto-occipital fasciculi (IFOF), uncinate fasciculi (UF), anterior thalamic radiation (ATR), inferior and superior longitudinal fasciculi (ILF and SLF), and forceps minor and major (FMi and FMa).

The tracts affected in our misophonia patients are involved in two cooperative brain functions that are of particular relevance to misophonia: social-emotional processing and attention towards emotionally salient information. The ILF and UF connect the amygdala to the occipital cortex, including the fusiform gyrus, and the OFC and frontal pole, respectively. Damage to the right UF impairs emotional empathy (Oishi et al., 2015), whereas high MD and RD in ILF and damage to the right IFOF have been associated with difficulty recognizing facial emotion (Genova et al., 2015, Philippi et al., 2009). Another tract connecting subcortical and cortical regions is the ATR, which connects the OFC and ATN. Interaction of these regions regulates automatic attentional capture by emotional stimuli and voluntary orienting towards task-relevant stimuli (Hartikainen et al., 2012). The anterior IFOF and SLF have likewise been linked to attention regulation and connect the frontal cortex to parietal and sensory (occipital and temporal, respectively) cortices (Sarubbo et al., 2013, Wu et al., 2016). Emotional disorders were found to share alterations in the SLF, which the authors specifically related to impaired perception of and attention to emotional information (Jenkins et al., 2016). The forceps minor and major connect the bilateral frontal and occipital cortices, respectively. Higher MD and RD in the FMi of ADHD patients correlated positively with inattention and negatively with executive function (Lawrence et al., 2013), whereas the FMa subserves visual processing. Lastly, the BCC connects bilateral superior frontal gyri. More specifically, the streamlines connected to a region also called the Premotor Ear-Eye Field (PEEF) that is specifically sensitive to complex auditory information and involved in gaze-shifting towards peripheral space based on information on sound spatial localization from auditory cortex (see Lanzilotto et al., 2013 for review).

The functional role of both tracts and connected brain areas implicated in our imaging study fits well with the neuropsychological profile of misophonia. Aberrant social-emotional processing, particularly recognition of facial emotion, is in line with the social nature of trigger sounds, which are generally produced by other humans, and previous studies finding greater right amygdala volume and abnormal activation and heightened resting-state connectivity of the fusiform cortex (Eijsker et al., 2021, Schröder et al., 2019), which processes stimuli of visual expertise such as human faces (Burns et al., 2019, Fox et al., 2009). Specifically, the latter was found with an independent component analysis in both fusiform and superior occipital cortex as part of the ventral attention network (Eijsker et al., 2021), which is involved in stimulus-driven attention. We interpreted this as sensory enhancement of trigger-related visual input, which we speculate could also be linked to the low MD in the FMi. WM abnormalities in attention-related networks is concordant with patients reporting heightened orientation towards aversive triggers, which activate the salience network, and their experiencing difficulties to shift their attention to other aspects of the environment (Kumar et al., 2017, Schröder et al., 2019). Indeed, a selective attention impairment has been found in the presence of symptom provoking sounds (da Silva and Sanchez 2018) and is in line with the aberrant N1 potential reflecting early automatic attentional processes (Schröder et al., 2014). Frank et al. (2020) likewise found impaired alerting attention in misophonia patients, which was irrespective of exposure to trigger sounds, suggesting a general attention deficit rather than a specific attentional bias.

Our modest sample size limits the generalizability of the results and power of the analyses, explaining why group effects with considerable effect sizes did not reach statistical significance. Also, Kumar et al. (2017) found WM alterations within vmPFC, which we did not find. This is likely due to the authors limiting their structural analysis to the areas that showed altered functional connectivity with the left anterior insula during symptom provocation. We, on the other hand, performed whole-brain exploratory analyses, which requires rigorous multiple comparison correction and therefore detects only strong and/or widespread effects. Still, replication of current results is warranted. Future studies might relate WM alterations in misophonia to differences in emotional reactivity, attentional functioning, and cognitive control and the effect of symptom-provocation hereon. Specifically, considering the amount of evidence implicating alterations in amygdala, fronto-parietal, and occipital areas, especially the fusiform cortices, we propose direct investigation of processing of trigger-related visual scenes in misophonia. This would primarily focus on human faces, considering the prevalence of nasal and oral misophonia triggers (Edelstein et al., 2013, Schröder et al., 2013).

In conclusion, we have found both macro and microstructural WM alterations in misophonia patients, implicating systems involved in social-emotional and attentional processing. These results provide further evidence for a neurobiological basis of misophonia. Yet additional research remains required to establish its biological basis that may provide the foundation for its classification as a discrete disorder.

## CRediT authorship contribution statement

**Nadine Eijsker:** Formal analysis, Writing - original draft, Project administration. **Arjan Schröder:** Conceptualization, Methodology, Writing - review & editing, Project administration. **Luka C. Liebrand:** Formal analysis, Resources, Writing - review & editing. **Dirk J.A. Smit:** Writing - review & editing, Supervision. **Guido Wingen:** Conceptualization, Methodology, Writing - review & editing, Supervision. **Damiaan Denys:** Conceptualization, Writing - review & editing, Supervision, Funding acquisition.

## Declaration of Competing Interest

The authors declare that they have no known competing financial interests or personal relationships that could have appeared to influence the work reported in this paper.
