# Supplementary material for: White matter abnormalities in misophonia
Source: Neuroimage Clin. 2021 Aug 21;32:102787. doi: 10.1016/j.nicl.2021.102787 (PMC8405911; doi:10.1016/j.nicl.2021.102787)
Supplement: Supplementary data 1 [file mmc1.docx]

| Subject scanning order | C C C M M C M C C M M C M M M M M M M C C M C C M M M M M M M M M C C C C M C C C C C C C M C C C |
| --- | --- |
| DTI acquisition parameters | 1 - 10 DTI with 64 gradient directions & 1 b=0 scan  11 – 13 DTI with 48 gradient directions & 1 b=0 scan  14 – 49 DTI with 64 gradient directions & 4 b=0 scans |
| Scan/experiment order | MPRAGE, resting-state, symptom provocation, DTI, B0, stop-signal task |
| Sample size calculation | While sample size was calculated based on the effect sizes of previous neuroimaging research on cognitive-behavioral therapy for anxiety disorders, the following article indicates that the sample size is sufficient for TBSS analysis: https://doi.org/10.1371/journal.pone.0067706 |

**Supplementary table 1. Additional study characteristics**

**Supplementary table 2. White matter volume differences between patients and controls.** Table displays all clusters with peak voxels with *p*-uncorrected < 0.001. *P*-values depicted in the table are FWE-corrected for multiple voxel-wise comparisons without additional Bonferroni correction. Bonferroni correction for number of contrasts (2) resulted in a significance threshold of *p*-FWE < 0.025. Peak voxels belonging to significant clusters are also highlighted.

| **Contrast** | **Region** | **voxels** | ***p*-FWE** | ***t*-stat** | ***Z*** | **MNI coordinates** |
| --- | --- | --- | --- | --- | --- | --- |
| **C < P** | 6% Inferior fronto-occipital fasciculus L, 6% Anterior thalamic radiation L, 2% Uncinate fasciculus L | 172 | 0.022 | 5.17 | 4.55 | -22 54 -6 |
|  | Genu of corpus callosum  15% Cingulum (cingulate gyrus) L | 3843 | 0.025 | 5.12 | 4.51 | -15 22 27 |
|  | 7% Anterior thalamic radiation L |  | 0.054 | 4.84 | 4.31 | -18 12 22 |
|  | Superior corona radiata L  1% Superior longitudinal fasciculus L |  | 0.087 | 4.66 | 4.17 | -20 0 32 |
|  |  | 3714 | 0.152 | 4.43 | 4.01 | 20 -4 34 |
|  |  |  | 0.254 | 4.21 | 3.84 | 22 8 27 |
|  |  |  | 0.400 | 3.99 | 3.67 | 18 15 16 |
|  |  | 242 | 0.226 | 4.26 | 3.88 | 27 -86 14 |
|  |  |  | 0.483 | 3.89 | 3.59 | 22 -74 10 |
|  |  | 317 | 0.255 | 4.21 | 3.84 | 24 -42 44 |
|  |  | 46 | 0.324 | 4.10 | 3.75 | 38 -66 6 |
|  |  | 116 | 0.329 | 4.09 | 3.75 | -20 -14 62 |
|  |  | 52 | 0.516 | 3.85 | 3.55 | 21 -32 10 |
|  |  |  | 0.686 | 3.65 | 3.39 | 26 -34 3 |
|  |  | 73 | 0.529 | 3.83 | 3.54 | -9 -50 12 |
|  |  |  | 0.793 | 3.52 | 3.28 | -12 -44 4 |
|  |  | 13 | 0.568 | 3.79 | 3.51 | -14 -48 40 |
|  |  | 41 | 0.615 | 3.74 | 3.46 | -44 -32 -2 |
|  |  | 190 | 0.633 | 3.71 | 3.45 | 16 -21 63 |
|  |  |  | 0.667 | 3.67 | 3.41 | 10 -22 57 |
|  |  | 35 | 0.830 | 3.47 | 3.24 | 18 -27 39 |
|  |  | 60 | 0.854 | 3.43 | 3.21 | 9 -45 20 |
|  |  | 10 | 0.871 | 3.40 | 3.19 | 18 -38 12 |
|  |  |  |  |  |  |  |
| **C > P** |  |  | 0.509 | 3.86 | 3.56 | 62 -24 8 |
|  |  |  | 0.697 | 3.64 | 3.38 | -16 -66 28 |

**Supplementary table 3. Correlations between clinical measures and white matter alterations in patients, corrected for age and sex.** No correlations reached the significance threshold (*p* < .018) provided by Dubey/Armitage-Parmar correction with Sankoh et al. (1997) modification for testing 3 clinical measures.

| **Measure** | **A-MISO-S**^a^ | | | **HAM-A**^b^ | | | **Anger (BPAQ)**^a^ | | |
| --- | --- | --- | --- | --- | --- | --- | --- | --- | --- |
|  | Correlation coefficient | *p*-value | Correlation coefficient | | *p*-value | Correlation coefficient | | *p*-value |  |
| WM ATR/IFOF | 0.06 | .808 | 0.04 | | .845 | -0.09 | | .699 |  |
| WM cingulum/GCC | -0.25 | .283 | 0.06 | | .795 | -0.17 | | .472 |  |
| Mean Diffusivity | -0.19 | .409 | -0.13 | | .954 | 0.09 | | .690 |  |

^a^ N = 23; ^b^ N = 24; A-MISO-S = Amsterdam Misophonia Scale; HAM-A = Hamilton Anxiety Rating Scale; BPAQ = Bush Perry Aggression Questionnaire; WM = White Matter; IFOF = Inferior Fronto-Occipital Fasciculus; ATR = Anterior Thalamic Radiation; GCC = Genu of Corpus Callosum
